# King Edward's Hospital Fund

**Published:** 1902-05-24

**Authors:** 


### The Institutional Workshop.

### KING EDWARD'S HOSPITAL FUND.

The letters from the Lord Mayor and Sir Francis Knollys, referred to in our last issue, page 112, which we append, will be read with interest. It is not a little remarkable that King Edward should place on one side all the proposals on the part of his subjects to present him with gifts of various kinds at the Coronation in favour of a monetary offering from them in aid of the London hospitals. King Edward's Hospital Fund has an income at the present time of about £50,000 a year. H.R.H. the Prince of Wales has received contributions which represent a permanent revenue of an additional £20,000 a year, and, if the gift to the King by his subjects at his coronation is sufficiently large to yield £50,000 a year in income, the object with which King Edward's Hospital Fund was originally established will have been largely fulfilled, and the London hospitals will be placed once and for all on a sound financial basis. In the letter which King Edward wrote dated February 5th, 1897, when the Prince of Wales's Fund for London was instituted, the King stated that the efforts of the Council would be concentrated upon an endeavour to secure from £100,000 to £150,000 in annual subscriptions from those who have not hitherto regularly contributed. If funds are therefore forthcoming on Coronation Day to the extent we have indicated, the revenue of King Edward's Hospital Fund of £120,000 per annum will be some £30,000 a year less than the maximum sum mentioned by the King in his original letter. The organisation of the League of Mercy, whereby small gifts of 1s. a year and upwards are obtained, is now, however, sufficiently established to warrant the confident expectation that within the next few years a revenue of from £25,000 to £30,000 per annum will be raised by its agency, and so the maximum sum originally asked for by the King will be forthcoming for the hospitals.

### A Friendly Lead by the Prince.

The Prince of Wales, the president of King Edward's Hospital Fund, by instituting a plan whereby he and each member of his family gives an annual subscription to the King's Fund has set an example which we hope will be widely followed. The King's Fund was instituted to commemorate the Diamond Jubilee of Queen Victoria, whose birthday was May 24th, and May 30th has been fixed officially for the celebration of the King's birthday. These dates will afford an opportunity to the head of every household, not only in London, but throughout the country, to follow the example of the Prince of Wales by enabling them on that day to receive from each member of their family and household a free gift to be sent in as their contribution to the Coronation gift. The Lord Mayor in his letter intimates that he has communicated with the mayors, and also with the bankers, not only in London, but in the provinces, so that each head of a household may take the contribution raised in the family circle and pay it in to the nearest bank or hand it to the Lord Mayor or mayor at the town hall adjacent to his place of residence. This is not an occasion where it is desirable to issue circulars inviting contributions to the Coronation gift, for the King in his letter states that nothing appeals more deeply to him than the needs of the hospitals, and asks for "such an increase to the permanent annual income of the King's Hospital Fund as will for ever testify to the loyal generosity evinced towards him by the people." We believe it is the first time in the history of our nation that King and people have thus been brought into direct personal contact by uniting to make adequate provision for the sick and suffering of all ranks. Every household, however humble or however exalted, has now an opportunity of testifying its loyalty and gratitude by contributing as a family to the Coronation gift. Pence will be as acceptable as pounds, and all donors have equal facilities and guarantees to secure that every penny of the money given shall go without deduction to the object for which it is contributed. If this simple plan of a family gift to the King from every household in each class of the community could be made generally known to the people, we have no doubt whatever that the whole of the money required for the hospitals would be forthcoming by Coronation Day. We hope in such an event that a book may be compiled in which will be entered the name of every family joining hands with the King, for in such a case the book of the Coronation gift could be handed down to future generations as a practical evidence of the unity of purpose which existed between King Edward and his people. In such circumstances this King's gift book would record the humanising influence of our hospitals upon all classes, and the growth of the spirit of benevolence and sympathy for others which has made the English people a power for good wherever their government has been established amongst the nations of the earth.

### London the Mecca of the Sick.

Some may feel at the first blush that the support of the London hospitals is a matter which only concerns residents in or visitors to London. In effect the benefits of the London hospitals extend to every part of the British Empire, for to them English people, wherever they may reside, are indebted to a great extent for the medical treatment and nursing care which are essentials in the day of sickness or accident. At the London hospitals are trained the larger proportion of the doctors and the nurses. To London from the provinces and from the British dependencies of the Empire come the most difficult and obscure cases of disease, because from their very nature the local medical attendant may advise that there, if anywhere, the latest scientific developments and the widest diagnostic experience are obtainable, and so a cure may result. These are some of the grounds upon which the King may well appeal to the intelligence of the people in the cause of the London hospitals, and they afford the best reasons why it is probable that the family gift idea will be gladly taken up throughout the country, and that it may even extend to the British dominions beyond the seas. The home is the true secret of the strength of the English people. No home is too exalted or too humble to fail to appreciate the claims of sickness upon the hale and the strong, and if the head of each household will bring his mind to bear upon the true inwardness of the motive which underlies the King's suggestion as to his Coronation gift, there can be no doubt that the majority of families will gladly welcome an opportunity to do their part to help the wayfaring man who falls out of the ranks in the battle of life. We hope that through the family gift King and people may have good cause to remember the coronation day of King Edward VII., for should his Majesty's wishes be fulfilled it will mark an epoch in the history of the English people, honourable to King and subjects alike, and far-reaching in consequences for good to both for all time.

The following are the letters of the Lord Mayor and Sir Francis Knollys :—

The Mansion House, London, E.C., May 12th, 1902.

Sir,—I have had the honour of receiving the accompanying letter from Sir Francis Knollys in reference to a meeting which I had decided to summon at the Mansion House on Monday, June 9th, in support of "King Edward's Hospital Fund for London." From this it will be observed that of all the many schemes put forward by his loyal and loving subjects for emphasising the great event of the Coronation, none appeals so warmly to his Majesty as the proposed gift to the King in the form of a further permanent endowment of this fund, which, the public will remember, was started by his Majesty himself, when Prince of Wales, five years ago, on the occasion of the Diamond Jubilee of Queen Victoria.

In anticipation of this interesting meeting, when practical steps will be taken to give effect to this important project, I venture to ask you to give publicity to the communication with which I have been honoured, and to invite all who are desirous of associating themselves with this mark of their affection for their Sovereign and his dynasty in a year so memorable in the annals of the Empire, to cordially respond, according to their ability, to the appeal about to be made.

It is, if I may respectfully say so, characteristic of His Majesty's kindness of heart and unfailing solicitude for the welfare of his subjects that he is graciously pleased to indicate that the proceeds of the Coronation Gift should be devoted to the alleviation of the sufferings of the sick poor in the capital of his dominions.

Donations to be announced at the meeting may be sent to me at the Mansion House; to the Bank of England; to Messrs. Prescott, Dimsdale, and Co., 50 Cornhill, or to the Hon. Secretary of "King Edward's Hospital Fund," 81 Cheapside, and will be gratefully acknowledged.

I shall also ask the London borough mayors and my brother bankers not only throughout the metropolis but the country generally to favour me by consenting to collect or receive contributions in enhancement of the Coronation Gift.—I am, Sir, your obedient servant,

Joseph C. Dimsdale, Lord Mayor.

The following is the text of Sir Francis Knollys' letter conveying the King's message of approval and sympathy :

Buckingham Palace, May 3, 1902.

Dear Lord Mayor,—I am commanded by the King to inform your lordship that His Majesty has learned with much satisfaction your proposal to summon a meeting of the citizens of London to collect funds for "King Edward VII. London Hospital Fund," with especial reference to the movement for presenting a Coronation Gift to His Majesty.

I am further desired to acquaint your lordship that among the many schemes which have been suggested for emphasising in a useful and benevolent form the great ceremonial of the Coronation, none appeals more deeply to His Majesty than that for augmenting the Hospital Fund which was instituted by him when Prince of Wales, in 1897, in commemoration of the Diamond Jubilee of the late illustrious Queen, and the welfare and prosperity of which the King has always watched with the greatest and most unfailing interest.

For these reasons I am commanded to say that the approaching meeting, and any practical steps resulting from it, has his Majesty's warmest sympathy, and that he trusts the movement may lead to such an increase to the permanent annual income of the fund as will for ever testify to the loyal generosity evinced towards him by the City of London.

I remain, dear Lord Mayor, yours very truly

Francis Knollys.

The Right Hon. the Lord Mayor, M.P.

